# Phagocytosis in Teleosts. Implications of the New Cells Involved

**DOI:** 10.3390/biology4040907

**Published:** 2015-12-04

**Authors:** María Ángeles Esteban, Alberto Cuesta, Elena Chaves-Pozo, José Meseguer

**Affiliations:** Department of Cell Biology and Histology, Faculty of Biology, University of Murcia, 30100 Murcia, Spain; E-Mails: alcuesta@um.es (A.C.); elena.chaves@mu.ieo.es (E.C.-P.); meseguer@um.es (J.M.)

**Keywords:** phagocytosis, immunity, teleosts

## Abstract

Phagocytosis is the process by which cells engulf some solid particles to form internal vesicles known as phagosomes. Phagocytosis is in fact a specific form of endocytosis involving the vesicular interiorization of particles. Phagocytosis is essentially a defensive reaction against infection and invasion of the body by foreign substances and, in the immune system, phagocytosis is a major mechanism used to remove pathogens and/or cell debris. For these reasons, phagocytosis in vertebrates has been recognized as a critical component of the innate and adaptive immune responses to pathogens. Furthermore, more recent studies have revealed that phagocytosis is also crucial for tissue homeostasis and remodeling. Professional phagocytes in teleosts are monocyte/macrophages, granulocytes and dendritic cells. Nevertheless, in recent years phagocytic properties have also been attributed to teleost lymphocytes and thrombocytes. The possible implications of such cells on this important biological process, new factors affecting phagocytosis, evasion of phagocytosis or new forms of phagocytosis will be considered and discussed.

## 1. Introduction

The immune system has developed success progressive complexity of basic functions that helped ancestral organisms to survive in their environment. The immune system responds to offensive using two crucial steps. In the first one, sensors detect the intruder. This intruder needs to interact with soluble or membrane-bound molecules present on the host. Some effectors elaborate a response and attack the intruder in the second step. The last three decades of study greatly improved our knowledge of how the immune system initiated and developed the refined immune mechanisms actually present on invertebrates and vertebrates. Among these mechanisms, phagocytosis is always seen as crucial.

Phagocytosis is the process by which a cell engulfs diverse particulate targets. This apparent simple process is the prime importance for all living organisms. Furthermore, evolution has provided phagocytes with a copious repertoire of molecules which are involved in this complex event. Phagocytosis was popularized at the end of the nineteenth century by the Russian embryologist Metchnikoff, who observed that amoeboid-like cells in transparent sea start larva contained ingested cells. He hypothesized that these cells would be able to recognize and internalize foreign material. Metchnikoff proved this idea in a simple experiment in which he observed that these cells moved toward and engulfed a thorn that he had introduced into a sea start larva. Based on these findings, he concluded that these supposed phagocytes were capable of ingestion and might play a key role in host defense and tissue homeostasis [1]. Since then, many of Metchnikoff’s ideas as to the origins of inflammation have been validated. What he could not have predicted is the huge heterogeneity and complexity of the biology of phagocytosis [2].

The present review focuses on the phagocytosis in fish although some comparisons with the most recent studies in other animal groups will be also considered. As usual, every time that new techniques have been used to study the phagocytosis, our idea about the process has been completed and even the ability to phagocytose for new cell types has been demonstrated. The earlier phagocytic assays in fish were carried out *in vivo* and afterwards, they were performed *in vitro* by using cell cultures or cell suspensions. To do the *in vitro* assays it was necessary firstly to isolate and characterize fish leucocytes. This step was difficult because most of the available studies focus on fish immune system concentrate mainly on lymphocytes [3]. *In vitro* assays make possible to know the significance and the influence of some conditions on the phagocytosis mechanisms (e.g., virulence of the bacteria, opsonization and leucocyte source) [4]. However, the use of cells from different organs and fish species, different target particles and phagocytosis protocols, difficulty the comparison of the obtained results. Furthermore, phagocytosis in fish had typically been studied using the population of glass and/or plastic adherent leucocytes derived mainly from the head kidney (the main hematopoietic organ), and to a lesser degree, from other lymphoid tissues or organs. In these assays, cells remained in suspension (as majority of granulocytes and B-cells, and dendritic cells) were excluded.

At the end of the nineties, flow cytometry started to be used to study fish phagocytosis process and new quantitative data about the phagocytic cell populations were obtained. Flow cytometry allows using of small samples, analyses a great cell numbers in a very short time and provides quantitative results. Furthermore, it permits a clear distinction between adhered and ingested particles (because some vital colorants have the property of quenching the fluorescence of the target particles remained outside the cells after the process of phagocytosis, while those particles inside the cells remain fluorescent because the colorants cannot penetrate the plasmatic membrane of living cells) and allows discrete cell evaluation [4]. In recent times, another great advance into the study of phagocytosis was due to the use of specific cell markers for different cell populations such as fish granulocytes or B cells [5,6,7]. Due to the reasons stated above, the ability of some cells to phagocytose in fish had remained unnoticed till recently.

Expert or professional phagocytes in fish are monocytes/macrophages and granulocytes. Nevertheless, in recent years phagocytic properties have also been attributed to teleost dendritic cells, lymphocytes and thrombocytes. The aim of the present review is to consider the implications of such “new” phagocytic cells avoiding the enumeration or the implications of the huge number of receptors and molecules involved in phagocytosis. Research in this area is expanding gradually and this review is intended to be just an introduction to the extensive literature available at present on this subject. Furthermore, some novel aspects of this old process will be discussed.

## 2. The Phagocytic Process

Phagocytosis is the most ancient and universal tool of defense against foreign material because unicellular eukaryotes phagocytose for food and defense. Amoebae already show mechanisms that allow recognition, internalization and destruction of foreign material [8]. In fact, amoebae and macrophages share similar phagocytic mechanisms such as recognition of the particle by cell surface receptors [9] and killing by oxygen radicals [10]. Apparently, the phagocytic machineries characterizing the amoeboid protozoans were inherited during the evolution towards innate immunity [11]. Although many mechanisms of immunity are common for invertebrates and vertebrates (phagocytosis, cytotoxicity, lectins, proteinases), others are only used in invertebrates (hemolymph clotting system, melanization) although the general plan on which they operate is realized in vertebrates as well [8].

Phagocytosis and macropinocytosis are fundamental processes of vertebrates that enable cells to test their environment, to remove pathogens and apoptotic bodies and, concomitantly, to offer immunoprotection. In fact, phagocytosis is the front-line mechanism by which the immune system eliminates most pathogenic microorganisms. Besides this, phagocytosis is an essential part of tissue homeostasis and remodeling and regulates the expression of different membrane components [12,13]. In vertebrates, phagocytosis appears to have developed from having a crucial role in innate immunity to sharing important functions in adaptive immunity [14,15]. Initially phagocytosis was defined as the process by which a cell internalized particles bigger than 0.5 micrometers. At present, the term phagocytosis is used to describe the process by which cells engulf particles such as bacteria, other microorganisms, aged red blood cells, foreign matter, *etc.*

The process of phagocytosis has been deeply studied in mammals and some aspects of this process have also been studied in fish, mainly in teleost and in those species of interest in aquaculture. The phagocytic process consists on a series of connected steps which include: (1) detection and recognition of the foreign particle; (2) attachment of the foreign particle to the phagocyte; (3) engulfment or internalization of the foreign particle into a vesicle called phagosome; (4) fusion of phagosome with a lysosome and formation of phagolysosome (degranulation of the phagocyte and maturation of compartment through endosomal fusion); (5) intracellular killing and digestion of the particle; (6) in the case of some phagocytes (e.g., macrophages and dendritic cells) egestion and antigen presentation.

The ultrastructure of the phagocytic process was studied for fish leucocytes by using bacteria cells as target particles and it was demonstrated that this process is variable as regards the number of bacteria engulfed per phagocyte and the time required for engulfment and digestion of each bacterium by the same phagocyte. This variability seems to provide greater host defence capacity [3,16]. The process of phagocytosis is known to be prompted by the interaction of surface molecules from the phagocytic target with receptors present on the phagocytic cells [14]. The surface of the phagocytes has many receptors that are able to recognize and decode their cognate ligands expressed on the surface of the phagocytic target and trigger engulfment. These receptors can directly recognize the particle or recognize targets which are coated with opsonic molecules. Initially these ligands were referred to as pathogen-associated molecular patterns (PAMPs). Actually, there is a necessity of including in these patterns the recognition commensal bacteria and apoptotic and necrotic cells. For this reason, a more inclusive term of “molecular pattern” (MP) was proposed [15]. Various kinds of pattern recognition receptors are involved in the identification of foreign factors in vertebrates and invertebrates [17]. Among such receptors of different evolutionary origins are scavenger receptors and Toll-like receptors. Also cytokine-like receptors and lectins may have originated in invertebrates independently from their vertebrate counterparts and may be unique to a particular taxonomic group.

Lysosomes fuse with phagosomes and form phagolysosomes. These are the vesicles where internalized microbes will be killed and degraded by a variety of lysosomal hydrolytic enzymes. Formation of phagosomes and phagolysosomes were described in fish phagocytes [3,7]. Moreover, phagocytosis is known to elicit several antimicrobial mechanisms. Arguably, the most important and well-known of these mechanisms involve the production of reactive oxygen (*i.e*., superoxide radicals) and nitrogen (*i.e*., nitric oxide) intermediates, which are known to kill ingested microbes contained in the phagolysosomes [18].

The molecular and cellular events that cause the binding of targets to a phagocyte and their engulfment into phagosomes have been extensively studied. More recent data suggest that the process of phagocytosis itself provides important information to myeloid phagocytes about the nature of the targets they are engulfing. Afterwards, this fact helps to tailor the appropriate inflammatory responses. How such information is acquired during phagocytosis and how it is processed to coordinate an immune response was recently reviewed in mammals [19]. However, the molecular mechanisms whereby different molecules translate the environmental cues into the complex and sophisticated responses that trigger the phagocytic process (e.g., phosphoinositide signaling) have to be established in fish [20].

## 3. Phagocytic Cells

While unicellular organisms use phagocytosis to obtain food, more complex metazoans have “professional” phagocytes (term used to described cells having high phagocytic ability and capacity) which act as an essential element of their immune system. In mammals and other vertebrates, including fish, professional phagocytes include polymorphonuclear cells (PMNs), monocytes and macrophages and dendritic cells (DCs) [18,21]. According to this terminology, non-professional phagocytes have more limited phagocytic properties than professional cells. Furthermore, non-professional phagocytes apparently lack the ability of producing microbicide oxygen and nitrogen products upon phagocytosis, and to secrete the cytokines characteristics of professional cells. Other cell types in mammals (*i.e*., epithelial cells, fibroblasts) have also been shown to be capable of engulfing particles, albeit with a much restricted capacity [21,22].

### 3.1. Macrophages and Granulocytes

Mammalian professional phagocytes derive from a common myeloid progenitor cell. Among these professional phagocytes, resident tissue macrophage populations are the first cells that encounter non-self-material, especially bacteria, and engulf and degrade them by using hydrolytic enzymes and oxidative attack. A variety of receptors on macrophages are used to detect infection [23]. Most of the studies done with fish phagocytes had shown that, like in mammals, monocytes, macrophages and neutrophils are the main phagocytic cells [18]. In fact, phagocytosis was considered the principal function to characterize or even identify macrophages in teleosts [24] and macrophages were described as the most active and avid phagocytes in fish [25,26]. These professional fish phagocytes differentially regulate pro-inflammatory and homeostatic responses *in vivo* [27]. Two other works focus on the fish macrophage and granulocyte functions and for this reason only a brief report focus on aspects considered of interest for this review will be presented.

During infection, macrophage lineage cells eliminate infiltrating pathogens through a battery of antimicrobial responses, where the efficacy of these innate immune responses is fundamental to immunological consequences. Due to the importance of these processes, many intracellular pathogens have evolved mechanisms to overcome macrophage defenses, using these immune cells as residences and dissemination strategies. Recent advances in the understanding of teleost macrophage antimicrobial responses and the strategies by which intracellular fish pathogens are able to avoid being killed by phagocytes have been reviewed [28]. It is known that pathogenic infections cause important detriments to both cultured and wild fish populations. It is of great interest to harvest greater understanding of fish phagocyte antimicrobial responses and the mechanisms by which aquatic pathogens are able to overcome these teleost macrophage barriers. Insights into the regulation of macrophage immunity of teleost species will lend to the development of more effective prophylaxis, at least, in farmed fish, as well as increase our knowledge of the evolution of these immune processes. 

Regarding granulocytes, three types of them have been identified in fish. It is known that mammalian neutrophils have a crucial role in the host tissue protection by killing and degradation of microorganisms and they are involved in inflammatory response. However, morphological heterogeneity of fish granulocytes, the lack of cell-specific surface markers and confusion of terms used to appoint the different types of granulocytes, makes difficult to establish which granulocyte types (neutrophils and/or eosinophils) are important phagocytes in fish [5,25,29,30,31]. It has long been known that neutrophils employ the two strategies previously described for macrophages to trap and kill invading pathogens: engulfment of microbes (phagocytosis) and secretion of antimicrobials (generation of reactive oxygen and nitrogen species). In 2004, a novel third function was identified. This new function was called neutrophil extracellular traps (NETs) [32]. NETs are networks of extracellular fibers (primarily composed of DNA from the cells) which bind pathogens. It is supposed that by the formation of NETs neutrophils are able of killing extracellular pathogens while minimizing damage to the host cells. NETs have also been shown to form within blood vessels during sepsis in mammals and intra-vascular NET formation is tightly controlled and is regulated by platelets [33]. Although the formation of NETs by fish granulocytes has started been studied [34] the implication of thrombocytes in the process has not been considered.

### 3.2. Dendritic Cells

Dendritic cells (DCs) are specialized antigen presenting cells that bridge innate and adaptive immunity in mammals. This link between the early innate immune system and the more evolutionarily recent adaptive immune system is of particular interest in fish, the oldest vertebrates to have both branches of the immunity. First studies on DCs on fish focus on provide some evidences that fish possessed cells homologous to mammalian DCs. For example, according to their morphological characteristics and non-adherent properties, DCs were identified in a long-term trout splenic culture [35]. Similarly, in spleen of nurse shark it was described a network present in the T cell rich areas forming by major histocompatibility complex (MHC) class II-positive cells [36]. The identification of Birbeck-like granules in cells of the gill epithelium and lymphoid tissue of salmonids was also attributed to the presence of DCs in such organs [37]. In Atlantic salmon it was obtained a cell line from cells having morphological characteristics of dendritic cell-like and with phagocytic properties [38]. More recently, some spleen and head kidney cells of rainbow trout stained with a CD207/langerin (expressed on specialized skin mammalian DCs called Langerhans cells) specific antibody [39]. The characterization of DCs in trout has been made by functional approaches. In fact, mammalian protocols for the generation of DCs were adapted to obtain cultures of highly mobile, non-adherent cells from trout hematopoietic tissue that had irregular membrane processes and expressed surface MHCII. The main function of MHC class II molecules is to present processed antigens, which are derived primarily from exogenous sources, to CD4(+) T-lymphocytes. Trout DCs have tree-like morphology, express dendritic cell markers, are able of phagocytose small particles, activate by toll-like receptor-ligands, and migrate *in vivo*. All these properties are hallmarks of mammalian DCs [40]. Furthermore, the identification of DCs in fish suggests that specialized antigen presenting cells evolved in concert with the emergence of adaptive immunity in lower vertebrates.

### 3.3. B Cells

Developmental and functional relationships between B cells and macrophages have long been recognized and several studies probed that mammalian malignant B cell lines can switch into macrophage-like cells, having the capacity to phagocytose large particles [41]. This switch from a lymphoid to a myeloid lineage gave rise to the term of “lineage switching” or “lineage infidelity”. Subsets of B lymphocytes and macrophages shared a closer lineage relationship than what the models of hematopoietic differentiation at the time predicted [42]. Thus, it would appear that professional phagocytosis was not restricted to cells of myeloid origin, as it can also be carried out by cells of lymphoid origin.

Until 2006, it was thought that primary B cells were unable to execute phagocytosis. Curiously, a study focus on fish B cells revealed, for the first time in vertebrates, the existence of B cell subsets with phagocytic and intracellular bactericidal capacities [7]. Afterwards, the phagocytic activity of B cells was also corroborated in amphibians, reptiles and more recently, in mammals. There are some evidences that this important innate capacity seems to be evolutionarily conserved in certain B cell subsets of vertebrates. Furthermore, Li and coworkers [7] demonstrated that phagocytosis by IgM+B cells was significantly improved by opsonization of bacteria with IgM or complement. These results seem to indicate that both Fc and complement receptors are present on fish phagocytic B cells. This study also quantify the contribution of this cell type into the phagocytic process because IgM+B cells represent the 62% and 20% of all the phagocytes present in rainbow trout blood and head-kidney, respectively.

In addition to rainbow trout, phagocytic IgM+B cells have also been demonstrated in other teleost fish species including catfish, cod and Atlantic salmon, which could suggest a differential contribution of phagocytes to the antimicrobial responses of these fish species. Catfish have also large amounts of phagocytic IgM+B cells in blood, while Atlantic salmon and cod contained significant numbers of phagocytic IgM+B cells in head kidney and blood [43]. Phagocytic IgM+B cells in salmon represented the vast majority of blood phagocytes whereas in cod they represented only 8% of these cells. Moreover, the phagocytic capacity of cod IgM+B cells was higher than that of salmon since over 70%–80% of the cod cells ingested three or more target beads in contrast to salmon in which that number was below 50% [43]. Thus it seems apparent that this phagocytic capacity is a general feature of all B cell lineages in teleost fish, although these results need to be confirmed in new studies carried out on other fish species. The evolutionary and functional relationships of fish and mammalian B cells, focusing mainly on the newly discovered roles of these cells in phagocytosis, intracellular killing and presentation of particulate antigen have been reviewed [44,45].

Similarly, B-lymphocytes of amphibians and reptiles have been described as phagocytic cells [7,46]. This fact has suggested a common ancestral function of such cells in innate immunity. Furthermore, in mammals, phagocytosis by non-myeloid leukocytes such as B-1 cells and gamma/delta-T-cells has also been reported [22]. This fact renovates our understanding on the phagocytic cell populations and its significance in both innate and adaptive immunity. 

Teleost fish B cells produce three different immunoglobulin (Ig) isotypes: IgM, IgD and IgT (the most recently identified). Whereas teleost IgM is mainly involved in systemic immunity, IgT appears to be an immunoglobulin specialized in mucosal immunity. Similarly, three major B cell lineages have been described in teleost, the most common lineage which co-expresses IgD and IgM and another one expressing either IgT or IgD [44]. Much remains to be investigated concerning the evolutionary origins and functional relationships of these fish B cell subsets and their implication on phagocytosis.

Particular interest could have to understand the rules related with the phagocytic properties of B cells on the gut. At present, it is known the importance of the relationships existing between intestinal microbiota and health existence. This situation requires the existence of physical separation between the microbiota and the host (e.g., mucus layer, secreted antimicrobials, the intestinal epithelium) and a very active control carry out by the immune system, in order to regulate the low number of microbes that could conquer the physical and chemical barrier present in the gut of vertebrates, even in healthy individuals. Recently, it has been reviewed how B-cell responses to members of the intestinal microbiota form a robust network with mucus, epithelial integrity, follicular helper T cells, innate immunity, and gut-associated lymphoid tissues to maintain host-microbiota mutualism in mammals [47]. To the best of our knowledge, these complex interactions established by B cells present in gut with the other elements play important roles in local immunity have not been studied in fish.

### 3.4. Thrombocytes

Thrombocytes are nucleated hemostatic blood cells of non-mammalian vertebrates. They are regarded as the functional equivalent of mammalian platelets (which are small anucleated cell fragments released from megakaryocytes) [48]. Although the function of platelets in the maintenance of hemostasis has been studied in great detail, more recent evidence has highlighted a central role for platelets in the host inflammatory and immune responses. Hemostasis contributes to fight microbial infection through blood vessel repair; in this process, platelets and thrombocytes play a triggering role in blood coagulation [49]. In addition to the known homeostatic functions, the possible roles of thrombocytes in immunity (including phagocytosis), have also been raised in different groups of animals, including fish [50,51,52]. 

Initial morphological studies demonstrated that bacteria act as a very potent aggregating agent for platelets and also promoting in these cells the secretion of different substances [53]. For many years, controversy exists as to whether thrombocytes could act as truly phagocytic cells in the inflammatory response. Furthermore, the data regarding their phagocytic ability were contradictory. The ontogeny of thrombocytes and platelets, the soluble factors derived from them involved in the inflammatory process, and their interaction with target particles were reviewed establishing a comparison between them [54]. Again, the study of the phagocytic properties of fish thrombocytes and their impact on pathogen clearance, have been controversial due to the difficulties of demonstrating definitive evidences with the available techniques [6,54]. Morphological observations of thrombocytes showed presumed phagocytosis of microorganisms. It was very easy to see (especially by transmission electron microscopy) the apparent internalization of target particles into thrombocytes [50,51]. However, these cells possess an extensive canalicular system open to the extracellular surface, which is a network of interconnected channels of the cell membrane to increase their cell surfaces and the release of various intracellular components [52,53]. This canalicular system can trap particles in a passive manner [54,55,56,57] although the particles will remain outside the cells; in other words, false images of phagocytosis could be observed by electron microscopy. Furthermore, enzymatic test presented inconsistent results in teleost thrombocytes [58]. Last year, it was demonstrated the potent active phagocytic activity of thrombocytes by using teleost (*Paralichthys olivaceus*) and amphibian (*Xenopus laevis*) models [59]. Furthermore, *ex vivo*, common carp thrombocytes were able to ingest live bacteria as well as latex beads (0.5–3 mm in diameter) in a manner dependent on actin polimerization and they were able of killing the ingested bacteria. Phagocytosis by thrombocytes was also enhanced by serum opsonization. Particle internalization led to phagolysosome fusion and killing of internalized bacteria, pointing to a strong capacity for microbe removal. Furthermore, they provide a deeper understanding of the potential immune function of mammalian platelets based on the conserved and vestigial functions [59]. These results regarding the characteristics of the phagocytosis process developed by fish thrombocyte are fairly comparable with those of classical phagocytes and remarking its functional significance in the innate immune defense of lower vertebrates. It has to be taken into account that, although these cells are able to interiorize a low number of particles, they are the second most abundant blood cells after the erythrocytes in fish [60]. Furthermore, phagocytic activity of head kidney thrombocytes has also been observed which is important because they could help to trigger the adaptive immune response. However, the relative contribution of the phagocytic thrombocytes to the total phagocytic capacity among the leucocyte pool warrants further consideration. It is necessary to study if fish thrombocytes are able to work as immune cells by initiating and modulating inflammatory and immune responses, or if they interact with endothelial cells and leucocytes directly by cell to cell contact and/or indirectly via secretion of soluble mediators as do platelets [61]. Furthermore, platelets store a multitude of immune-associated molecules in their granules and, upon activation (in response to various factors such as thrombin, chemokines or microbial toxins) they express adhesive and immune receptors (e.g., P-selectin, CD40 ligand, and Toll-like receptors) on their surface, and release soluble mediators such as chemokines, cytokines, and antimicrobial peptides [62]. New studies are needed to understand the implication of fish thrombocytes in the innate and acquired immunity or to know if fish thrombocytes also participate in the host immune response by directly killing infected cells, as do platelets in mammals [61]. The application of new techniques (e.g., intravital imaging) will allow to visualize the cellular behavior *in vivo* or to see the diverse cell types involved in vascular anti-bacterial immunity in fish.

## 4. Factors Affecting Phagocytosis

The susceptibility of fish to disease is partly dependent on their environment, in particular on water temperature. The influence of different environmental factors on phagocytosis was studied some years ago. Influence of temperature on fish phagocytosis was described and total immune competence in teleosts at low environmental temperatures was discussed [63]. Nevertheless, comparison between different works studying phagocytosis by fish cells is difficult due to the wide variability on methodological methods applied. It has also been taken into account that results about phagocytosis are very variable because numerous factors can affect this process, as it was previously mentioned. Among them, animal fish species, source of the cells, different methodologies applied, the choice of the target particle or the cell-to particle ratio. Actually, new factors, such as hypoxia, circadian rhythms or ageing, affecting phagocytosis in fish start to be considered. Hypoxia is an important factor for adaptation to cellular stress and the transcriptional regulation of cell metabolism. It modulates the function of phagocytic cells by stimulating surface receptors such as scavenger receptors, toll like receptors and their downstream signaling cascades [64]. In response to hypoxia, innate immune modifiers are up-regulated through pathways involving the key immune response master regulator nuclear factor-κB leading to the modulation of inflammatory cytokines. 

Regarding circadian rhythms, they are endogenous 24-h variations found in virtually all physiological processes. These circadian rhythms are generated by circadian clocks. Most immune cells express circadian clock genes and present a wide array of genes expressed with a circadian rhythm. This has deep consequences on cellular functions, including daily circadian rhythms of cellular functions, such as phagocytosis. The possible implications of these data for human health start to be obtained [65]. Similarly, age-related impairments in leucocyte function are likely to have important consequences for the health of the older population. It has been reported that ageing in macrophages impacts on many processes including phagocytosis [66,67]. In fish, most studies on immune system focus on their initial steps of growth in order to establish proper development pattern of the immune activities. However, those studies have still not been carried out on mature or brood fish. Furthermore, initial studies on fish phagocytes focus on the influence of several intrinsic or extrinsic factors on the process but, as it was previously mentioned, those assays were carried out mainly on macrophages. For this reasons, new studies focus on the effect of different variables on other phagocytic cell types are still needed.

## 5. Evasion of Phagocytosis

Professional phagocytes mediate processes ranging from phagocytosis to tissue homeostasis. This is possible because they effectively engulf and eliminate invading microorganisms. To survive this assault, pathogens have developed an array of countermeasures aimed at avoiding detection (strategies to avoid recognition and uptake by host cell or altered host signaling to promote invasion, manipulation of host cell cytoskeleton.) impairing signaling, or paralyzing the machinery that underlies phagocytosis. Some facultative and obligate intracellular bacteria have evolved ways to evade or even exploit autophagy [68]. Furthermore, certain pathogens benefit from attaching to, entering, or traversing host cells to establish and spread infection [69]. Other intracellular bacterial pathogens drive the formation of host “pseudo-organelles” that facilitate their replication, survival, or latency. The formation and maintenance of these bacteria-containing vacuoles are dependent on the bacteria's ability to commandeer the host's intracellular membrane system (mainly dynamic compartments involved in exo-/endocytic membrane traffic). Additionally, bacterial survival or proliferation inside the vesicles could be augmented by host membrane transport processes subverted by secreted bacterial factors, which facilitate the acquisition of membrane sources and nutrients [68]. For example, *Leishmania* has evolved ingenious ways to adapt to life in the macrophage. New proteins have recently been found to disrupt processes ranging from antigen cross-presentation to nuclear pore dynamics. Furthermore, *Leishmania* sabotages key metabolic and signaling pathways and induces DNA methylation to turn off genes controlling microbicidal pathways. These novel findings highlight the creative attack employed by *Leishmania* to subvert macrophage function [70]. Diverse strategies used by different pathogenic bacteria to prevent the bacteria-containing vacuoles from being destroyed via the endolysosomal pathway have been studied in mammals; although there are some similar studies in fish [28] there are still many questions to be resolved in this interesting area.

## 6. Some New Aspects of Phagocytosis Still Not Considered in Fish 

It seems reasonable to think that regarding new aspects to be considered regarding fish phagocytes, many new molecules involved will be described. As an example, recently connexin 43 and pannexin 1 have been found in mammalian immune cells. Furthermore, while gap junctional communication has been demonstrated between immune cells, hemichannels have been implicated in many cellular functions. Among the functions involved as being connexin dependent and pannexin dependent are cell migration, phagocytosis, antigen presentation, T-cell reactivity and B-cell responses [71].

Besides the study of new molecules involved in phagocytosis, many other aspects should be also considered because constantly, new particles to be interiorized, new physiological sceneries and new evasion mechanisms of phagocytosis by pathogens are described. Only some of them will be underline in the present review.

The rapid increase in the use of nanotechnology products is enlarging the presence of nanoparticles (NPs) (metal, metal-oxide and carbon-based) in the aquatic environment. Although most NP types do not exhibit or have very low direct toxicity, could display masked sub-lethal immunotoxic effects. Effects of the NPs on innate immune system, such as effects on phagocytes, might be suitable for screening for immunotoxicity because these cells mediate both innate and adaptive immune responses. Recently, the effects of NPs used in consumer and medical applications (gold, silver, titanium dioxide, silica dioxide, zinc oxide, and carbon nanotubes) and polystyrene NPs on the immune system have been reviewed [72,73]. Effects in animal exposures through different routes are compared to the effects on isolated phagocytes. In general, NPs appear to induce a specific immunotoxic pattern consisting of the induction of inflammation in normal animals and aggravation of pathologies in disease models. It is expected that the evaluation of particle action on several phagocyte functions *in vitro* may provide an indication on the potency of the particles to induce immunotoxicity *in vivo* and to know the health risks of NPs.

Animal bodies has a high number of cell turnover (e.g., human bodies collectively turn over about 200 to 300 billion cells every day) as a fundamental part of development (embryonic and postnatal), as well as habitual tissue homeostasis. This process involves the induction of programmed cell death in specific cells and the recognition and removal of dying cells by phagocytes [74]. The phagocytic cells involved in such processes in fish have not been still studied. Similarly, the broad immunologic roles of autophagy (literally “self-eating”) could be explored. For example, one of the best-appreciated manifestations of autophagy is protection against microbial invasion, but this is by no means limited to direct elimination of intracellular pathogens and includes a stratified array of nearly all principal immunologic processes [75]. In addition to its vital homeostatic role, autophagy has been implicated in many different cellular processes such as cell apoptosis, inflammation, pathogen clearance, and antigen presentation and thereby has been linked to a variety of human disorders [76]. The intimately linked molecular mechanisms that help govern the autophagic pathway and macrophage innate immune responses in mammals have been revised [77]. 

New forms of phagocytosis studied at present only in mammals, will be studied in fish. Some examples are described in the next paragraphs. Phagoptosis (known also as primary phagocytosis) is the term chosen to describe a form of cell death caused by phagocytosis of viable cells, resulting in their destruction. It is provoked by exposure of “eat-me” signals and/or loss of “don’t-eat-me” signals by viable cells, causing their phagocytosis by phagocytes. Phagoptosis mediates turnover of cells (being one of the main forms of cell death in the body), defends against pathogens and regulates inflammation and immunity. Furthermore, it is not yet established if phagoptosis contributes or not to pathology [78]. *Entamoeba histolytica* was named “histolytica” for its ability to destroy host tissues. This amoeba, after attaching to host cells, bites off and ingests distinct host cell fragments, and that this contributes to cell killing. This process, termed “amoebic trogocytosis” (trogo-, “nibble”), interplays with phagocytosis, or whole cell ingestion, in this organism. “Nibbling” processes have also been described not only in other microbes but also in multicellular organisms [79]. Inflammatory caspases play a central role in innate immunity by responding to cytosolic signals and initiating a twofold response that trigger a form of lytic, programmed cell death called pyroptosis. Pyroptosis removes the replication niche of intracellular pathogens, making them susceptible to phagocytosis and killing by a secondary phagocyte [80].

Macrophages working along with neutrophils and dendritic cells are one of the key effector cells initiating and directing the host reaction to pathogenic organisms and resolving subsequent responses. Extracellular traps (ETs) are a relatively novel strategy of host defense involving expulsion of nuclear material and embedded proteins from immune cells to immobilize and kill bacteria, fungi, and viruses [81].

## 7. Conclusions 

For many decades the phenomenon of phagocytosis has been of prime interest to medical scientists and immunologists as an important factor in the defense of the host in infectious diseases. Furthermore, phagocytosis has also held the interest of biologists in general as a manifestation of cellular activity. Though we know many aspects of this fascinating process, whenever we head into one of them it opens up a whole range of unknown possibilities that are worth exploring. It seems that we know only just the beginning of a very complex reality that simply called phagocytosis.

## References

[1] Metchnikoff E. (1905). Immunity in the Infectious Diseases.

[2] Stuart L.M., Ezekowitz R.A. (2005). Phagocytosis: Elegant complexity. Immunity.

[3] Esteban M.A., Meseguer J. (1994). Phagocytic defence mechanism in sea bass (*Dicentrarchus labrax* L.): An ultrastructural study. Anat. Rec..

[4] Esteban M.A., Meseguer J. (1997). Factors influencing phagocytic response of macrophages from the sea bass (*Dicentrarchus labrax* L.): An ultrastructural and quantitative study. Anat. Rec..

[5] Sepulcre M.P., Pelegrín P., Mulero V., Meseguer J. (2002). Characterization of gilthead seabream acidophilic granulocytes by a monoclonal antibody unequivocally points to their involvement in fish phagocytic response. Cell Tissue Res..

[6] Mulero I., Sepulcre P., Roca F., Meseguer J., García-Ayala A., Mulero V. (2008). Characterization of macrophages from the bony fish gilthead seabream using an antibody against the macrophage colony-stimulating factor receptor. Dev. Comp. Immunol..

[7] Li J., Barreda D.R., Zhang Y.A., Boshra H., Gelman A.E., Lapatra S., Tort L., Sunyer O., Lapatra S., Tort L., Sunyer O. (2006). B lymphocytes from early vertebrates have potent phagocytic and microbicidal abilities. Nat. Immunol..

[8] Dzik J.M. (2010). The ancestry and cumulative evolution of immune reactions. Acta Biochim. Pol..

[9] Allen P.G., Dawidowicz E.A. (1990). Phagocytosis in *Acanthamoeba*: I. A mannose receptor is responsible for the binding and phagocytosis of yeast. J. Cell Physiol..

[10] Davies B., Chattings L.S., Edwards S.W. (1991). Superoxide generation during phagocytosis by *Acanthamoeba castellanii*: similarities to the respiratory burst of immune phagocytes. J. Gen. Microbiol..

[11] Sillo A., Bloomfield G., Balest A., Balbo A., Pergolizzi B., Peracino B., Skelton J., Ivens A., Bozzaro S. (2008). Genome-wide transcriptional changes induced by phagocytosis or growth on bacteria in *Dictyostelium*. BMC Genomics.

[12] Flannagan R.S., Jaumouillé V., Grinstein S. (2012). The cell biology of phagocytosis. Annu. Rev. Pathol..

[13] Pedrera I.M., Collazos M.E., Ortega E., Barriga C. (1992). *In vitro* study of the phagocytic processes in splenic granulocyte of the tench (*Tinca tinca*, L.). Dev. Comp. Immunol..

[14] Desjardins M., Houde M., Gagnon E. (2005). Phagocytosis: The convoluted way from nutrition to adaptive immunity. Immunol. Rev..

[15] Moretti J., Blander J.M. (2014). Insights into phagocytosis-coupled activation of pattern recognition receptors and inflammasomes. Curr. Opin. Immunol..

[16] Secombes C.J., Fletcher I.C. (1992). The role of phagocytes in the protective mechanisms of fish. Annu. Rev. Fish Dis..

[17] Akira S. (2006). TLR signaling. Curr. Top Microbiol. Immunol..

[18] Neumann N.F., Stafford J.L., Barreda D., Ainsworth A.J., Belosevic M. (2001). Antimicrobial mechanisms of fish phagocytes and their role in host defense. Dev. Comp. Immunol..

[19] Underhill D.M., Goodridge H.S. (2012). Information processing during phagocytosis. Nat. Rev. Immunol..

[20] Levin R., Grinstein S., Schlam D. (2015). Phosphoinositides in phagocytosis and macropinocytosis. Biochim. Biophys. Acta.

[21] Rabinovitch M. (1995). Professional and non-professional phagocytes: An introduction. Trends Cell Biol..

[22] Wu Y., Wu W., Wong W.M., Ward E., Thrasher A.J., Goldblatt D., Osman M., Digard P., Canaday D.H., Gustafsson K. (2009). Human gamma delta T cells: A lymphoid lineage cell capable of professional phagocytosis. J. Immunol..

[23] Zhang L., Wang C.C. (2014). Inflammatory response of macrophages in infection. Hepatobiliary Pancreat Dis. Int..

[24] Braun-Nesje R., Kaplan G., Seljelid R. (1982). Rainbow trout macrophages *in vitro*: Morphology and phagocytic activity. Dev. Comp. Immunol..

[25] Rowley A.F., Hunt T.C., Page M., Mainwaring G., Rowley A.F., Ratcliffe N.A. (1988). Fish. Vertebrate Blood Cell.

[26] Enane N.A., Frenkel K., O’Connor J.M., Squibb K.S., Zelikoff J.T. (1993). Biological markers of macrophage activation: Applications for fish phagocytes. Immunology.

[27] Rieger A.M., Konowalchuk J.D., Grayfer L., Katzenback B.A., Havixbeck J.J., Kiemele M.D., Belosevic M., Barreda D.R. (2012). Fish and mammalian phagocytes differentially regulate pro-inflammatory and homeostatic responses *in vivo*. PLoS ONE.

[28] Grayfer L., Hodgkinson J.W., Belosevic M. (2014). Antimicrobial responses of teleost phagocytes and innate immune evasion strategies of intracellular bacteria. Dev. Comp. Immunol..

[29] Ainsworth A.J. (1992). Fish granulocytes: Morphology, distribution and function. Annu. Rev. Fish Dis..

[30] Zapata A.G., Chib A., Varas A., Iwama G., Najanishi T. (1996). Cells and tissue of the immune system of fish. The Fish Immune System.

[31] Rønneseth A., Wergeland H.I., Pettersen E.F. (2007). Neutrophils and B-cells in Atlantic cod (*Gadus morhua* L.). Fish Shellfish Immunol..

[32] Brinkmann V., Reichard U., Goosmann C., Fauler B., Uhlemann Y., Weiss D.S., Weinrauch Y., Zychlinsky A. (2004). Neutrophil extracellular traps kill bacteria. Science.

[33] Clark S.R., Ma A.C., Tavener A.S., McDonald B., Goodarzi Z., Kelly M.M., Patel K.D., Chakrabarti S., McAvoy E., Sinclair G.D. (2007). Platelet TLR4 activates neutrophil extracellular traps to ensnare bacteria in endotoxemic and septic blood. Nature Med..

[34] Brogden G., Krimmling T., Adamek M., Naim H.Y., Steinhagen D., von Köckritz-Blickwede M. (2014). The effect of β-glucan on formation and functionality of neutrophil extracellular traps in carp (*Cyprinus carpio* L.). Dev. Comp. Immunol..

[35] Ganassin R.C., Bols N. (1996). Development of long-term rainbow trout spleen cultures that are haemopoietic and produce dendritic cells. Fish Shellfish Immunol..

[36] Rumfelt L.L., McKinney E.C., Taylor E., Flajnik M.F. (2002). The development of primary and secondary lymphoid tissues in the nurse shark *Ginglymostoma cirratum*: B-cell zones precede dendritic cell immigration and T-cell zone formation during ontogeny of the spleen. Scand. J. Immunol..

[37] Lovy J., Wright G.M., Speare D.J. (2008). Comparative cellular morphology suggesting the existence of resident dendritic cells within immune organs of salmonids. Anat. Rec..

[38] Pettersen E.F., Ingerslev H.C., Stavang V., Egenberg M., Wergeland H.I. (2008). A highly phagocytic cell line TO from Atlantic salmon is CD83 positive and MCSFR negative, indicating a dendritic-like cell type. Fish Shellfish Immunol..

[39] Lovy J., Savidant G.P., Speare D.J., Wright G.M. (2009). Langerin/CD207 positive dendritic-like cells in the haemopoietic tissues of salmonids. Fish Shellfish Immunol..

[40] Bassity E., Clark T.G. (2012). Functional identification of dendritic cells in the teleost model, rainbow trout (*Oncorhynchus mykiss*). PLoS ONE.

[41] Hanecak R., Zovich D.C., Pattengale P.K., Fan H. (1989). Differentiation *in vitro* of a leukemia virus-induced B-cell lymphoma into macrophages. Mol. Cell Biol..

[42] Borrello M.A., Phipps R.P. (1996). The B/macrophage cell: An elusive link between CD5+ B lymphocytes and macrophages. Immunol. Today.

[43] Overland H.S., Pettersen E.F., Ronneseth A., Wergeland H.I. (2010). Phagocytosis by B-cells and neutrophils in Atlantic salmon (*Salmo salar* L.) and Atlantic cod (*Gadus morhua* L.). Fish Shellfish Immunol..

[44] Sunyer J.O. (2012). Evolutionary and functional relationships of B cells from fish and mammals: Insights into their novel roles in phagocytosis and presentation of particulate antigen. Infect. Disord. Drug Targets.

[45] Parra D., Takizawa F., Sunyer J.O. (2013). Evolution of B cell immunity. Annu. Rev. Anim. Biosci..

[46] Zimmerman L.M., Vogel L.A., Edwards K.A., Bowden R.M. (2010). Phagocytic B cells in a reptile. Biol. Lett..

[47] Slack E., Balmer M.L., Macpherson A.J. (2014). B cells as a critical node in the microbiota-host immune system network. Immunol. Rev..

[48] Belamarich F.A., Fusari M.H., Shepro D., Kien M. (1966). *In vitro* studies of aggregation of non-mammalian thrombocytes. Nature.

[49] Kim S., Radhakrishnan U.P., Rajpurohit S.K., Kulkarni V., Jagadeeswaran P. (2010). Vivo-Morpholino knockdown of alpha IIb: A novel approach to inhibit thrombocyte function in adult zebrafish. Blood Cells Mol. Dis..

[50] Grecchi R., Saliba A.M., Mariano M. (1980). Morphological changes, surface receptors and phagocytic potential of fowl mononuclear phagocytes and thrombocytes *in vivo* and *in vitro*. J. Pathol..

[51] Stosik M., Deptuła W., Travnicek M., Baldy-Chudzik K. (2002). Phagocytic and bactericidal activity of blood thrombocytes in carps (*Cyprinus carpio*). Vet. Med..

[52] Wigley P., Hulme S.D., Barrow P.A. (1999). Phagocytic andoxidative burst activity of chicken thrombocytes to *Salmonella*, *Escherichia coli* and other bacteria. Avian Pathol..

[53] White J.G., Clawson C.C. (1981). Effects of large latex particle uptake of the surface connected canalicular system of blood platelets: A freeze-fracture and cytochemical study. Ultrastruct. Pathol..

[54] Meseguer J., Esteban M.A., Rodríguez A. (2002). Are thrombocytes and platelets true phagocytes?. Microsc. Res. Tech..

[55] Nakayasu C., Yoshitomi T., Oyamatsu T., Okamoto N., Ikeda Y. (1997). Separation of carp (*Cyprinus carpio* L.) thrombocytes by using a monoclonal antibody, and their aggregation by collagen. Vet. Immunol. Immunopathol..

[56] White J.G. (1972). Uptake of latex particles by blood platelets: Phagocytosis or sequestration?. Am. J. Pathol..

[57] White J.G. (2005). Platelets are covercytes, not phagocytes: Uptake of bacteria involves channels of the open canalicular system. Platelets.

[58] Daimon T., Gotoh Y., Kawai K., Uchida K. (1985). Ultrastructural distribution of peroxidase in thrombocytes of mammals and submammals. Histochemistry.

[59] Nagasawa T., Nakayasu C., Rieger A.M., Barreda D.R., Somamoto T., Nakao M. (2014). Phagocytosis by thrombocytes is a conserved innate immune mechanism in lower vertebrates. Front Immunol..

[60] Imagawa T., Hashimoto Y., Kitagawa H., Kon Y., Kudo N., Sugimura M. (1989). Morphology of blood cells in carp (*Cyprinus carpio* L.). Jpn. J. Vet. Sci..

[61] Ferdous F., Scott T.R. (2015). A comparative examination of thrombocyte/platelet immunity. Immunol. Lett..

[62] Tamagawa-Mineoka R. (2015). Important roles of platelets as immune cells in the skin. J. Dermatol. Sci..

[63] Le Morvan C., Troutaud D., Deschaux P. (1998). Differential effects of temperature on specific and nonspecific immune defences in fish. J. Exp. Biol..

[64] Mishra K.P., Ganju L., Singh S.B. (2015). Hypoxia modulates innate immune factors: A review. Int. Immunopharmacol..

[65] Labrecque N., Cermakian N. (2015). Circadian clocks in the immune system. J. Biol. Rhythm..

[66] Linehan E., Fitzgerald D.C. (2015). Ageing and the immune system: focus on macrophages. Eur. J. Microbiol. Immunol..

[67] Parker G.A., Picut C.A. (2012). Immune functioning in non lymphoid organs: The liver. Toxicol. Pathol..

[68] Tang B.L. (2015). Bacteria-containing vacuoles: Subversion of cellular membrane traffic and Autophagy. Crit. Rev. Eukaryot Gene Expr..

[69] Sarantis H., Grinstein S. (2012). Subversion of phagocytosis for pathogen survival. Cell Host Microbe.

[70] Arango Duque G., Descoteaux A. (2015). A *Leishmania* survival in the macrophage: Where the ends justify the means. Curr. Opin. Microbiol..

[71] Glass A.M., Snyder E.G., Taffet S.M. (2015). Connexins and pannexins in the immune system and lymphatic organs. Cell Mol. Life Sci..

[72] Jovanović B., Palić D. (2012). Immunotoxicology of non-functionalized engineered nanoparticles in aquatic organisms with special emphasis on fish—Review of current knowledge, gap identification, and call for further research. Aquat. Toxicol..

[73] Fröhlich E. (2015). Value of phagocyte function screening for immunotoxicity of nanoparticles *in vivo*. Int. J. Nanomed..

[74] Arandjelovic S., Ravichandran K.S. (2015). Phagocytosis of apoptotic cells in homeostasis. Nat. Immunol..

[75] Deretic V., Kimura T., Timmins G., Moseley P., Chauhan S., Mandell M. (2015). Immunologic manifestations of autophagy. J. Clin. Invest..

[76] Yu T., Zuber J., Li J. (2015). Targeting autophagy in skin diseases. J. Mol. Med..

[77] Vural A., Kehrl J.H. (2014). Autophagy in macrophages: Impacting inflammation and bacterial infection. Scientifica.

[78] Brown G.C., Neher J.J. (2012). Eaten alive! Cell death by primary phagocytosis: “Phagoptosis”. Trends Biochem. Sci..

[79] Ralston K.S. (2015). Chew on this: Amoebic trogocytosis and host cell killing by *Entamoeba histolytica*. Trends Parasitol..

[80] Jorgensen I., Miao E.A. (2015). Pyroptotic cell death defends against intracellular pathogens. Immunol. Rev..

[81] Boe D.M., Curtis B.J., Chen M.M., Ippolito J.A., Kovacs E.J. (2015). Extracellular traps and macrophages: New roles for the versatile phagocyte. J. Leukoc Biol..

